# Peripheral GABA Signaling in Metabolic Adaptation and Maladaptation

**DOI:** 10.3390/ijms27167141

**Published:** 2026-08-09

**Authors:** Tolulope Peter Saliu, Adedeji O. Adetunji, Johnson O. Ogunsile, Hannah O. Popoola, Chinyere Mary-Cynthia Ikele, Sierra N. Miller, Kelly Oriakhi, Fernando Diaz, Stephen P. Karaganis

**Affiliations:** 1 Department of Life, Earth and Environmental Sciences, West Texas A&M University, Canyon, TX 79016, USA; snmiller@wtamu.edu (S.N.M.); fdiaz@wtamu.edu (F.D.); skaraganis@wtamu.edu (S.P.K.); 2 Department of Agriculture, University of Arkansas at Pine Bluff, Pine Bluff, AR 71602, USA; 3 Molecular Targets and Therapeutics Center, Institute of Virology, Helmholtz Munich, 85764 Neuherberg, Germany; ogunsilejohnson@gmail.com; 4 Division of Clinical Pharmacology, Faculty of Medicine IV, Ludwig-Maximilians-University of Munich (LMU), 80337 Munich, Germany; 5 Department of Chemistry & Biochemistry, Miami University, Oxford, OH 45056, USA; hanpopoola@gmail.com; 6 Department of Biology, New Mexico State University, Las Cruces, NM 88003, USA; chinyere@nmsu.edu; 7 Department of Pharmacology and Nutritional Sciences, College of Medicine, University of Kentucky, Lexington, KY 40536, USA; kelly.oriakhi@uky.edu

**Keywords:** γ-aminobutyric acid (GABA), peripheral GABA signaling, metabolic adaptation, metabolic maladaptation, obesity, type 2 diabetes mellitus, insulin resistance, inter-organ communication

## Abstract

Peripheral γ-aminobutyric acid (GABA) signaling is emerging as a context-dependent contributor to metabolic regulation. Long recognized as the principal inhibitory neurotransmitter in the central nervous system, GABA’s role as an important signal in peripheral tissues is recently getting attention. This expanded view raises a central question: why do GABA-sensitive pathways support regulation in some metabolic settings, yet reinforce dysfunction in others? In obesity and type 2 diabetes mellitus (T2DM), nutrient excess, insulin resistance, and chronic inflammation remodel the cellular environments in which GABA is produced, sensed, and metabolized. As a result, GABA signaling may shift from adaptive regulation that maintains tissue function to compensatory responses that attempt to limit metabolic stress, and ultimately to maladaptive outputs that reinforce disease progression. Here, we review the biochemical basis, sources, receptor systems, and extracellular regulation of peripheral GABA signaling. We then examine how GABA-sensitive pathways are organized across metabolic tissues and remodeled in obesity and T2DM, with emphasis on islet endocrine dysfunction, hepatic GABA output, and adipose–immune–microbiota interactions. We further consider how this framework informs pathway-specific therapeutic strategies and the barriers to translation. We propose that peripheral GABA signaling is neither inherently protective nor harmful. Its metabolic consequence depends on the source of GABA, the responding tissue environment, and the stage of metabolic disease.

## 1. Introduction

γ-Aminobutyric acid (GABA) is classically defined as the principal inhibitory neurotransmitter in the central nervous system (CNS) [1]. However, this CNS-centered definition does not fully account for growing evidence that GABA-dependent pathways operate across peripheral tissues and contribute to metabolic regulation [2,3,4,5]. In these extra-neural compartments, GABA functions as more than a neurotransmitter outside the nervous system. It is synthesized, metabolized, and detected within tissue microenvironments that are shaped by endocrine, immune, hepatic, nutritional, and microbial signals [2,3,4,5,6]. This broader biology raises a central question for metabolism: how should GABA be interpreted when the same molecule can participate in intracellular metabolism, local signaling, and systemic exposure?

This question is especially relevant in obesity and type 2 diabetes mellitus (T2DM). These disorders not only alter circulating glucose, insulin, and lipid levels, but also remodel the tissues that interpret metabolic signals [7,8,9,10]. Nutrient excess, impaired insulin action, ectopic lipid accumulation, and low-grade inflammation alter the cellular environments in which signaling pathways are engaged [7,8,9,10]. A signal that supports local regulation under one physiological condition may therefore have different functional consequences when tissue architecture, inflammatory tone, or metabolic demand changes. Peripheral GABA signaling exemplifies this context dependence because its effects are determined by the tissue in which it is produced, the cell populations that can sense it, and the disease state in which the pathway is engaged. The expansion of GABA biology beyond the nervous system has created both opportunity and ambiguity. GABA-related enzymes, receptors, and transport systems have been described in endocrine, hepatic, immune, and gut-associated compartments [2,3,4,5,11]. Dietary and microbial sources may also contribute to peripheral GABA availability [5,6,12,13]. Yet the presence of GABA or its signaling machinery does not, by itself, define function. Within one tissue microenvironment, GABA may support adaptive endocrine or metabolic regulation; within another, it may reflect compensation or contribute to maladaptive output. This context dependence is the central challenge in interpreting peripheral GABA biology in metabolic disease.

In this narrative Review, we examine GABA as a peripheral metabolic signal whose function depends on its biochemical source, tissue organization, and disease state. We first outline the biochemical features that determine GABA availability and signaling potential. We then consider how GABA-sensitive pathways are organized across metabolic tissues and how obesity and T2DM remodel these pathways. Particular attention is given to islet endocrine dysfunction, hepatic GABA output, and adipose–immune–microbiota interactions. This framework is used to distinguish between GABA pathways that may preserve metabolic adaptation from those that may require therapeutic restraint or redirection.

## 2. GABA Biochemistry and Peripheral Signaling

**Figure 1:**
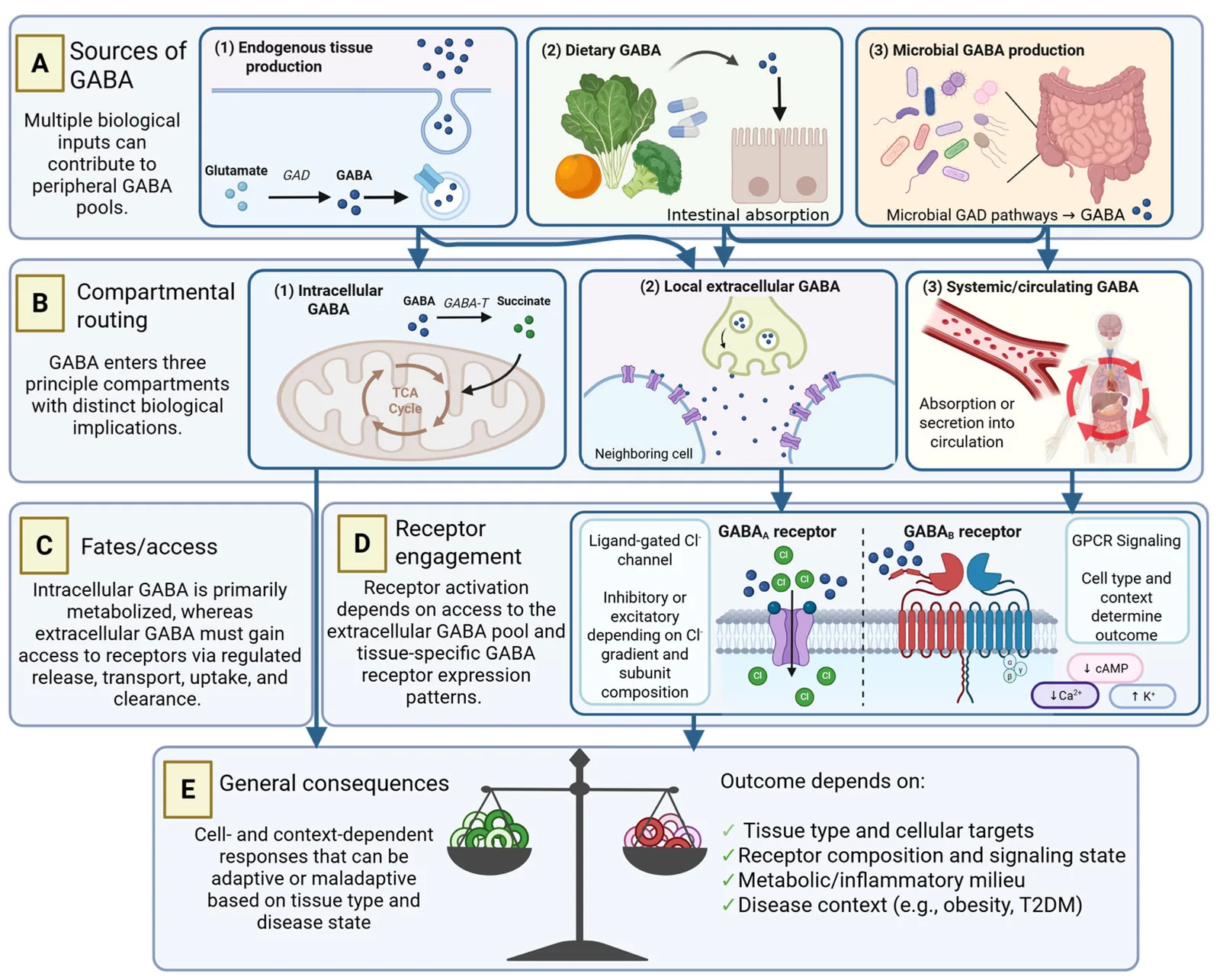
**Compartmental organization of peripheral GABA sources, fate, and receptor access.** The schematic summarizes peripheral γ-aminobutyric acid (GABA) biology across five interconnected levels: source, compartmental routing, metabolic fate and extracellular access, receptor engagement, and functional consequence. (**A**) Peripheral GABA may arise from endogenous tissue production through glutamate decarboxylase (GAD), dietary intake followed by intestinal absorption, or microbial GAD-dependent production within the gut. (**B**) These sources may contribute to intracellular, local extracellular, or systemic/circulating GABA pools, which have distinct biological implications. Intracellular GABA can enter the GABA shunt, in which GABA transaminase (GABA-T) generates succinic semialdehyde that is subsequently converted to succinate and enters tricarboxylic acid (TCA) cycle metabolism. Local extracellular GABA may act on neighboring receptor-competent cells, whereas absorbed or secreted GABA may contribute to systemic exposure. (**C**) The availability of extracellular GABA is regulated by release, transport, uptake, circulation, and clearance. (**D**) Receptor engagement occurs primarily through ionotropic GABA_A__AA receptors and metabotropic GABA_B__BB receptors. GABA_A__AA-receptor responses depend on chloride gradients and receptor subunit composition, whereas GABA_B__BB receptors signal through G proteins to regulate cyclic AMP, calcium, and potassium signaling. (**E**) The resulting effects are cell- and context-dependent and are shaped by tissue type, cellular target, receptor composition, metabolic and inflammatory conditions, and disease state. Peripheral GABA may therefore function as an intracellular metabolic intermediate, a local extracellular ligand, or an indicator of systemic exposure, with consequences that may support metabolic adaptation or contribute to maladaptation. GAD, glutamate decarboxylase; GABA-T, GABA transaminase; GPCR, G-protein-coupled receptor; TCA, tricarboxylic acid; T2DM, type 2 diabetes mellitus.

GABA is a small, four-carbon, non-proteinogenic amino acid with metabolic and signaling functions [14]. Its biological role is shaped by the route of production, the compartment in which it accumulates, and the mechanisms that regulate its release, receptor access, and clearance [14,15,16,17]. In the CNS, these processes support inhibitory neurotransmission through coordinated synthesis, vesicular release, receptor activation, transporter-mediated uptake, and degradation [14,15,16,17]. Outside the CNS, GABA biology is not confined to a synaptic neurotransmitter architecture. Host tissues, dietary intake, and microbial metabolism can each contribute to peripheral GABA pools, placing the same molecule in biochemical environments that differ in concentration, duration, and accessibility to responding cells [2,5,6,18]. These source-to-fate relationships give peripheral GABA three distinct biological roles. First, intracellular GABA can function as a metabolic intermediate through the GABA shunt. Second, GABA that reaches the extracellular space can act as a ligand for receptors on competent responding cells. Third, GABA detected in blood, tissue homogenates, intestinal contents, or fecal samples may indicate peripheral exposure or abundance, but not receptor engagement. These roles are biologically related but should not be treated as equivalent (Figure 1).

### 2.1. Synthesis and Metabolic Fate

In mammalian cells, GABA is produced mainly through the decarboxylation of L-glutamate by glutamate decarboxylase enzymes [15,19]. This pyridoxal 5′-phosphate-dependent reaction converts glutamate into GABA and provides the principal route of mammalian GABA synthesis [15,19]. The two major mammalian glutamate decarboxylase isoforms are GAD67 and GAD65, encoded by *GAD1* and *GAD2*, respectively [15,19,20]. Both enzymes catalyze the same reaction, but their regulation and intracellular distribution differ [15,19,20]. GAD67 is generally associated with constitutive cytosolic GABA production, whereas GAD65 is more dynamically regulated and has been linked to vesicular or secretion-associated GABA availability in neural systems [19,20]. These differences are best defined in neurons. In peripheral tissues, they are most useful as a biochemical guide, indicating that the same synthetic pathway may feed intracellular metabolism, regulated release, or both.

The fate of newly synthesized GABA depends on how the producing cell handles it after synthesis. One major intracellular route is the GABA shunt, in which GABA transaminase converts GABA to succinic semialdehyde [14]. Succinic semialdehyde is then oxidized to succinate, linking GABA turnover to the tricarboxylic acid cycle [14]. Through this route, GABA contributes to mitochondrial substrate handling and cellular energy metabolism rather than functioning only as a signaling molecule [14]. GABA retained within the intracellular compartment can contribute to metabolic pathways, whereas GABA released or transported into the extracellular space can become available for receptor-mediated signaling. Thus, intracellular GABA abundance may reflect synthesis, uptake, storage, or metabolism, whereas receptor-mediated signaling requires extracellular access to a competent target cell.

### 2.2. Transport, Extracellular Access, and Receptor Engagement

Peripheral GABA signaling begins with a spatial constraint: GABA produced within one cell must reach an extracellular site where a responding cell can sense it. Plasma membrane GABA transporters help establish this local pool by controlling transmembrane GABA flux, with net movement determined by ionic gradients and cellular state [16]. Vesicular transport adds directionality by linking intracellular storage to regulated release [2,21]. In neurons, these mechanisms operate within synapses, where release, receptor activation, and uptake are spatially constrained. Peripheral tissues lack this defined synaptic architecture; therefore, the GABA available to responding cells is governed by tissue structure, cellular proximity, and the balance between release and uptake.

Once a local extracellular GABA pool is formed, its effect is determined by the receptor landscape and physiological state of the responding cell. GABA mainly signals through ionotropic GABA_A_ receptors and metabotropic GABA_B_ receptors [17,22,23,24]. GABA_A_ receptors form ligand-gated chloride channels, and their effects depend on receptor subunit composition, extracellular GABA concentration, chloride gradients, and cation–chloride cotransporter activity [17,22,25]. Consequently, GABA_A_ receptor activation is not uniformly inhibitory in peripheral tissues. In pancreatic α-cells, GABA_A_ receptor signaling contributes to the restraint of glucagon secretion, whereas activation of GABA_A_ receptors in human β-cells can be depolarizing and support insulin secretion [26,27,28,29]. GABA_B_ receptors function as heteromeric assemblies containing GABA_B1_ and GABA_B2_ subunits and signal through Gi/o-coupled pathways [23,24]. Their metabolic consequences are also tissue-dependent. In brown adipose tissue, GABA_B_ receptor signaling has been linked to tissue dysfunction and systemic metabolic impairment during obesity [30]. Together, these examples show that peripheral GABA receptor function cannot be inferred from receptor expression alone, but must be interpreted in relation to cell identity, chloride homeostasis, and intracellular signaling context.

This spatial organization makes bulk GABA measurements difficult to interpret in isolation. GABA detected in serum, tissue homogenates, or fecal samples can indicate exposure or abundance, but it may not represent the receptor-accessible pool within a defined tissue niche. In peripheral GABA signaling, the biological response is determined not only by the amount of GABA present, but also by its localization, the route of extracellular availability, and the cell populations competent to respond.

### 2.3. Sources of Peripheral GABA

Peripheral GABA originates from three broad but biologically distinct sources: mammalian tissues, dietary intake, and microbial metabolism. The origin of a GABA pool influences not only where the molecule is generated, but also how it becomes available to host tissues and how its biological effects should be interpreted. Distinguishing these sources is therefore essential for separating GABA production or exposure from evidence of functional signaling.

#### 2.3.1. Host-Derived GABA

Several peripheral cell populations produce host-derived GABA, although its synthesis, handling, and release vary among tissues. In pancreatic β-cells, glutamate decarboxylase isoforms support GABA synthesis, and the resulting pool is distributed between cytosolic and vesicular compartments and may also enter intracellular metabolism [31]. Endothelial cells have likewise been shown to synthesize and release GABA [32], whereas immune cells possess machinery for GABA synthesis and catabolism [33]. Collectively, these findings establish that endogenous GABA production extends beyond the nervous system and is organized according to cell-specific physiology. However, identification of GABA or its synthetic machinery establishes source potential rather than biological function. Functional attribution requires evidence that locally produced GABA becomes accessible to responsive cells and contributes to a measurable tissue effect.

#### 2.3.2. Dietary GABA

Dietary GABA constitutes an exogenous source whose biological relevance depends on gastrointestinal transit and intestinal absorption. Human pharmacokinetic studies demonstrate that orally administered GABA is absorbed and produces measurable increases in circulating concentrations [6,12], while Caco-2 epithelial models provide complementary evidence of transport across an intestinal barrier [34]. Together, these findings establish that dietary GABA is not confined to the gut lumen but can contribute to peripheral exposure. Following absorption, this exogenous pool may also enter downstream metabolic pathways rather than functioning solely as an extracellular ligand. In mice, oral GABA increases skeletal muscle homocarnosine, indicating incorporation into GABA-derived peptide metabolism [35]. Dietary GABA should therefore be viewed as a bioavailable source that increases peripheral exposure and can enter downstream metabolic pathways after absorption. Increased systemic availability, however, does not identify the tissue in which GABA acts or establish engagement of a defined receptor pathway.

#### 2.3.3. Microbiota-Derived GABA

Microbiota-derived GABA is generated within the intestinal ecosystem through bacterial glutamate decarboxylase pathways. GABA production has been demonstrated in selected intestinal strains of *Lactobacillus* and *Bifidobacterium* [36] and across multiple human intestinal *Bacteroides* species [37]. This capacity is strain dependent and shaped by substrate availability and the metabolic conditions of the gut environment [18,36,37]. Experimental manipulation of the microbiota further indicates that gut ecology can modify GABA-related pools and associated host responses. Administration of GABA-producing lactobacilli alters intestinal GABA concentrations and metabolic phenotypes in mice [5]. In contrast, prebiotic and enzyme-based interventions increase gut and brain GABA concentrations without identifying the precise microbial source or the distal tissue access route [13]. In a hepatic ischemia–reperfusion model, metformin-associated microbiota remodeling identified microbiota-derived GABA as a mediator of the observed tissue-protective response [38]. These studies establish microbial GABA production and demonstrate biological activity in selected experimental settings. Whether comparable source-to-function relationships operate across metabolic tissues in obesity and T2DM remains less well defined. The biological significance of microbiota-derived GABA depends on how its site of production is coupled to access, signaling, and response within host tissues.

## 3. Organ-Specific Actions of Peripheral GABA in Metabolism

**Figure 2:**
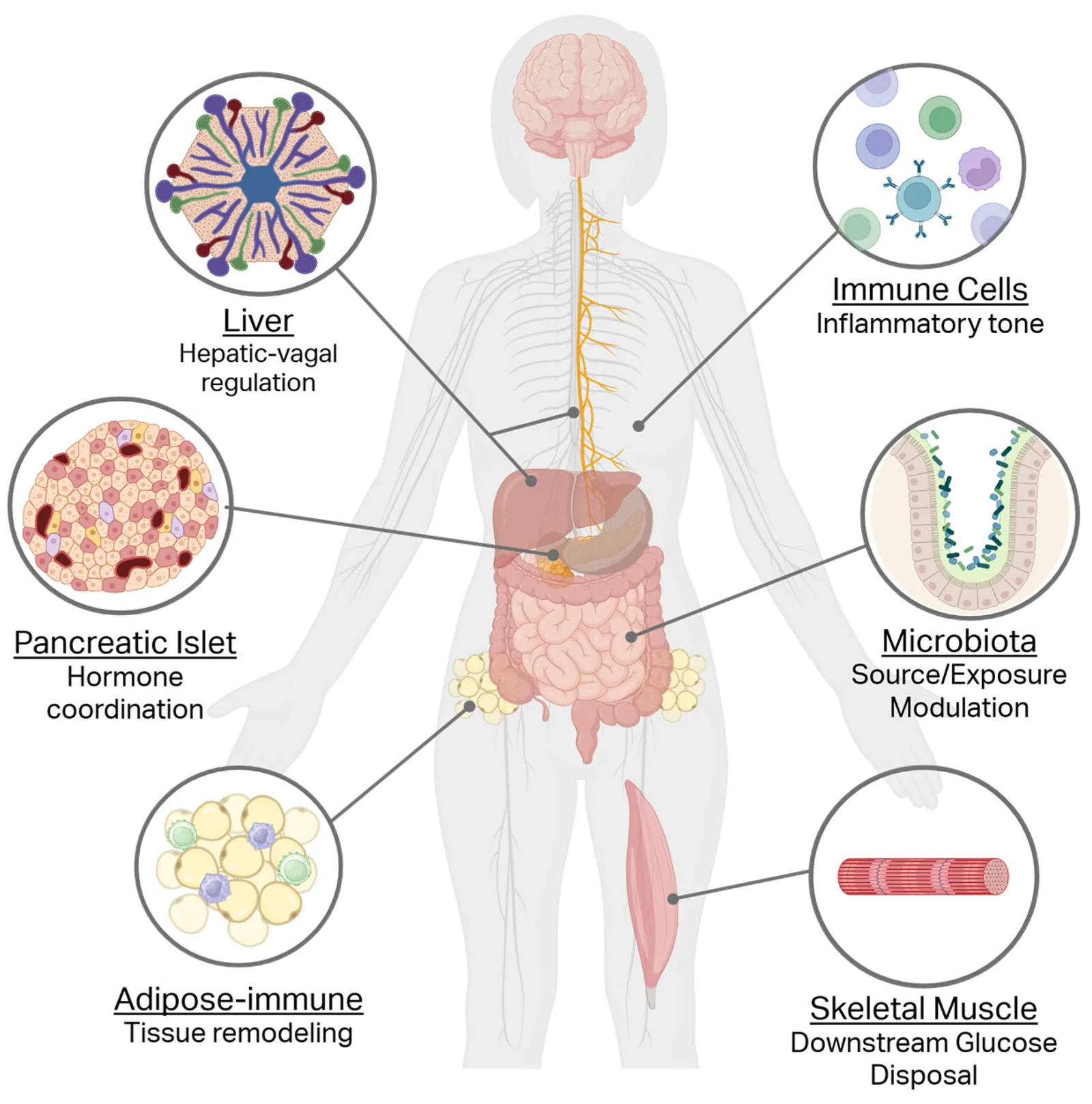
**Tissue-dependent outputs of peripheral GABA signaling.** Peripheral GABA signaling aligns with distinct tissue-specific functions across metabolic organs and related compartments. 1, Pancreatic islet: GABA contributes to intra-islet endocrine coordination and restraint of glucagon output. 2, Liver: hepatocyte-derived GABA is linked to hepatic–vagal regulation of insulin-related physiology. 3, Adipose–immune tissue: GABA-associated responses are linked to adipose tissue remodeling and immune–stromal communication. 4, Immune cells: leukocyte GABA-sensitive pathways are associated with inflammatory tone and immune effector outputs. 5, Microbiota: gut microbial metabolism can modify GABA source and peripheral exposure. 6, Skeletal muscle: skeletal muscle is shown as an insulin-sensitive downstream tissue in which GABA-associated effects may be reflected through glucose disposal, GABA-derived peptide metabolism or repair-associated responses. GABA, γ-aminobutyric acid.

Fuel homeostasis is distributed across tissues that store, release, consume, and respond to nutrients [10,39]. These processes depend on local communication within individual organs and on signals transmitted through the circulation and neural/autonomic pathways [40,41,42]. Peripheral GABA intersects with this organization at multiple points, linking the same molecule to distinct metabolic outputs across tissues [3,27,42,43]. Yet how GABA-handling pathways are organized across metabolic organs remains incompletely resolved. Emerging evidence suggests that GABA is not coupled to a single common peripheral response but instead aligns with the organ-specific physiological function of the tissue in which it acts [3,27,42,43]. This organ-level organization is illustrated in Figure 2, while representative experimental evidence for tissue-specific GABA action is summarized in Table 1.

### 3.1. Pancreatic Islets and Endocrine Coordination

The endocrine pancreas maintains glucose homeostasis through reciprocal regulation of insulin and glucagon secretion. Although these hormones act systemically, their secretion is coordinated locally within the islets of Langerhans [40]. In these endocrine microorgans, β-cells, α-cells, and other endocrine cell types communicate via paracrine signals that modulate hormone release in response to nutrient status [40]. Evidence from rodent islet models identifies GABA as a β-cell-associated paracrine signal that restrains α-cell glucagon output [26,27]. β-cell-derived GABA suppresses glucagon secretion from neighboring α-cells, while intra-islet insulin enhances α-cell GABA_A_-receptor signaling through Akt-dependent receptor trafficking [26,27]. Together, these mechanisms define a β-cell-to-α-cell pathway through which β-cell activity restrains glucagon release and supports coordinated islet hormone output [26,27].

The cellular organization of the rat islet further supports this local pathway. GABA transporter 3 is present in both α-cells and β-cells, whereas the vesicular inhibitory amino acid transporter localizes to secretory granules and synaptic-like microvesicles [2]. This distribution places GABA transport and vesicular storage machinery within compartments involved in local hormone release, providing a cellular basis for regulated intra-islet GABA availability [2].

During glucose-stimulated β-cell activity, GABA is released into the islet microenvironment alongside insulin secretion, although the two signals may originate from distinct intracellular pools [47]. Once released, GABA activates GABA_A_ receptors on neighboring endocrine cells. In α-cells, the chloride gradient generally favors chloride influx and membrane hyperpolarization, thereby reducing electrical activity and glucagon secretion [48]. Human β-cells maintain a different chloride equilibrium, such that GABA_A_-receptor activation can promote chloride efflux and membrane depolarization. This response increases β-cell excitability and can support insulin secretion [28,29]. Thus, the effect of intra-islet GABA is determined not only by receptor expression but also by the chloride gradient and identity of the responding endocrine cell.

Human islet studies further identify distinct effects of GABA on β-cell physiology. Beyond its effects on β-cell excitability and insulin secretion, GABA has also been implicated in β-cell maintenance. Experimental studies indicate that GABA can promote human β-cell proliferation and modulate glucose homeostasis [49]. This evidence supports a potential role in β-cell maintenance, although the proliferative response remains model-dependent and, by itself, does not establish durable restoration of coordinated endocrine function. Collectively, current evidence supports three aspects of islet GABA biology. Glucagon restraint is demonstrated predominantly in rodent models, whereas studies in human islets support modulation of β-cell secretory activity. Evidence for β-cell proliferation remains more context-dependent and is currently limited to experimental systems.

### 3.2. Liver and Hepatic–Vagal Regulation

The liver maintains systemic fuel homeostasis by coordinating glucose production, glycogen turnover and lipid handling across fasting and postprandial states [50,51,52]. These functions are governed by circulating substrates and hormones and are further integrated with autonomic circuits that communicate hepatic metabolic status to the central nervous system [41]. Within this neuro-metabolic interface, hepatocyte-derived GABA has emerged as a signal linking the metabolic and electrical state of the liver to hepatic vagal afferent nerve activity [42]. The operation of this pathway therefore depends on the mechanisms that regulate GABA movement across the hepatocyte membrane and its availability at the hepatic–vagal interface.

Extracellular hepatic GABA availability is regulated, at least in part, by hepatocyte membrane potential and electrogenic solute carrier 6 transporters. Hepatocytes express BGT1/SLC6A12, GAT2/SLC6A13, TauT/SLC6A6 and CRT/SLC6A8 [42]. In liver-slice preparations, inhibition of BGT1 or GAT2 increases extracellular GABA, consistent with predominant roles for these transporters in GABA uptake. TauT and CRT have been proposed as potential routes of GABA efflux, although the transporter responsible for GABA export from hepatocytes has not been definitively identified [42]. The activity and direction of these electrogenic transporters depend on transmembrane ion gradients, allowing changes in hepatocyte membrane potential to shift the balance between GABA uptake and release. Accordingly, hepatocyte depolarization increases hepatic GABA release, whereas hyperpolarization restrains transporter-mediated release [42]. Membrane-potential-sensitive regulation of extracellular GABA may therefore couple hepatocyte state to hepatic neural signaling.

Once available at the hepatic–vagal interface, extracellular GABA suppresses hepatic vagal afferent nerve firing [42]. The ability of the GABA_A_-receptor agonist muscimol to reproduce this response provides functional evidence for a GABA_A_-receptor-sensitive step in the pathway [42]. However, the receptor subunits involved and the identity of the receptor-bearing cell population remain unresolved. It is therefore unclear whether hepatocyte-derived GABA acts directly on vagal afferent endings or indirectly through another hepatic cell type. The downstream neural circuitry through which altered hepatic afferent activity influences systemic insulin physiology is likewise incompletely defined. Current evidence thus supports a membrane-potential-sensitive hepatic GABA–vagal signaling pathway, although its transporter, receptor and circuit-level organization remains to be established.

### 3.3. Adipose Tissue and Tissue Remodeling

Adipose tissue contributes to systemic energy balance through lipid storage, lipid mobilization, and endocrine secretion [53]. Beyond these canonical functions, white adipose depots display marked cellular plasticity. In response to nutritional, endocrine, and tissue-derived cues, these depots can acquire beige adipocyte features, including thermogenic capacity and increased energy expenditure [54]. This plasticity provides a basis for understanding how peripheral signals may influence adipose tissue function beyond lipid storage alone. Experimental evidence has associated GABA exposure with changes in adipose tissue plasticity, including white-to-beige remodeling of inguinal white adipose tissue and increased expression of thermogenic markers [43]. Complementary studies in adipocyte models also link GABA exposure to changes in adipogenic, lipogenic, and thermogenic programs [55]. These findings identify adipose tissue as a GABA-responsive metabolic compartment, although the direct responding cell population and receptor pathway remain incompletely defined.

### 3.4. Peripheral Immune Cells and Inflammatory Tone

Immune cells support tissue surveillance, repair, homeostasis, and inflammatory regulation through cytokine production, chemokine signaling, migration, and context-dependent changes in activation state [56,57]. In metabolic organs, these immune outputs influence the local environment in which parenchymal and stromal populations maintain tissue function [56]. This organization also identifies the immune compartment as a site through which peripheral GABA signaling can act, because leukocytes express GABA-related enzymes, transporters, and receptors and exhibit GABA-responsive inflammatory and effector outputs [3,44,45,58,59,60]. In human peripheral blood mononuclear cells and CD4^+^ T cells, GABA reduces the release of inflammatory cytokines [3]. In macrophages, GABA-associated pathways regulate IL-1β production, linking GABA transport and signaling to inflammasome-related responses [44,45]. GABAergic signaling has also been associated with immune-cell migration and activation state, including dendritic-cell hypermigration during infection and B-cell-derived GABA induction of IL-10-producing macrophages [59,60]. Collectively, these findings establish direct GABA responsiveness across several immune-cell populations, with outcomes shaped by leukocyte lineage, transporter or receptor repertoire, and activation state [3,44,45,59,60]. The metabolic significance of these immune effects is less clearly defined. In studies reporting improved glucose homeostasis or reduced adipose inflammation, immune changes accompany these metabolic improvements [61,62]. However, direct evidence that a specific GABA-responsive immune population mediates these improvements remains limited. Establishing causality will require cell-specific manipulation of GABA receptors, transporters, or metabolic pathways, together with tissue-level and whole-body metabolic measurements.

### 3.5. Skeletal Muscle as a Downstream Tissue in Peripheral GABA Biology

Skeletal muscle is a major site of insulin-stimulated glucose disposal in humans and an important site of glycogen synthesis [10,39]. Accordingly, changes in systemic insulin action are expressed prominently through muscle glucose handling. Within peripheral GABA biology, the strongest evidence for skeletal-muscle involvement arises from pathways that alter whole-body insulin physiology rather than from a clearly defined local GABA signaling mechanism. The relationship between hepatic GABA and skeletal muscle illustrates this organization. In obese mice, liver-derived GABA alters insulin-related physiology through hepatic–vagal signaling [42], whereas liver-directed suppression of GABA transaminase increases skeletal-muscle glucose uptake [4]. These findings indicate that altered hepatic GABA output can influence muscle glucose disposal and place skeletal muscle downstream of a liver-derived GABA pathway.

Evidence from oral exposure studies further indicates that skeletal muscle participates in the metabolic handling and tissue-level consequences of peripheral GABA. In mice, dietary GABA is incorporated into skeletal-muscle homocarnosine, whereas oral GABA has been associated with improved muscle regeneration in diabetic animals [35,46]. These observations extend the relevance of skeletal muscle beyond glucose disposal to GABA-derived metabolism and repair-associated responses. They do not, however, establish that local GABA signaling directly regulates muscle glucose uptake, insulin signaling, or mitochondrial metabolism.

Peripheral GABA is therefore linked to skeletal muscle principally through systemic metabolic regulation and downstream tissue responses rather than through a well-defined local signaling pathway. More broadly, the consequences of peripheral GABA depend on the physiological function and regulatory state of the tissue in which the pathway is engaged. This context becomes particularly important in obesity and type 2 diabetes, where endocrine, hepatic, adipose, immune, and skeletal-muscle physiology are extensively remodeled [4,7,8,63].

## 4. Metabolic Remodeling of Peripheral GABA Signaling in Obesity and Type 2 Diabetes

**Figure 3:**
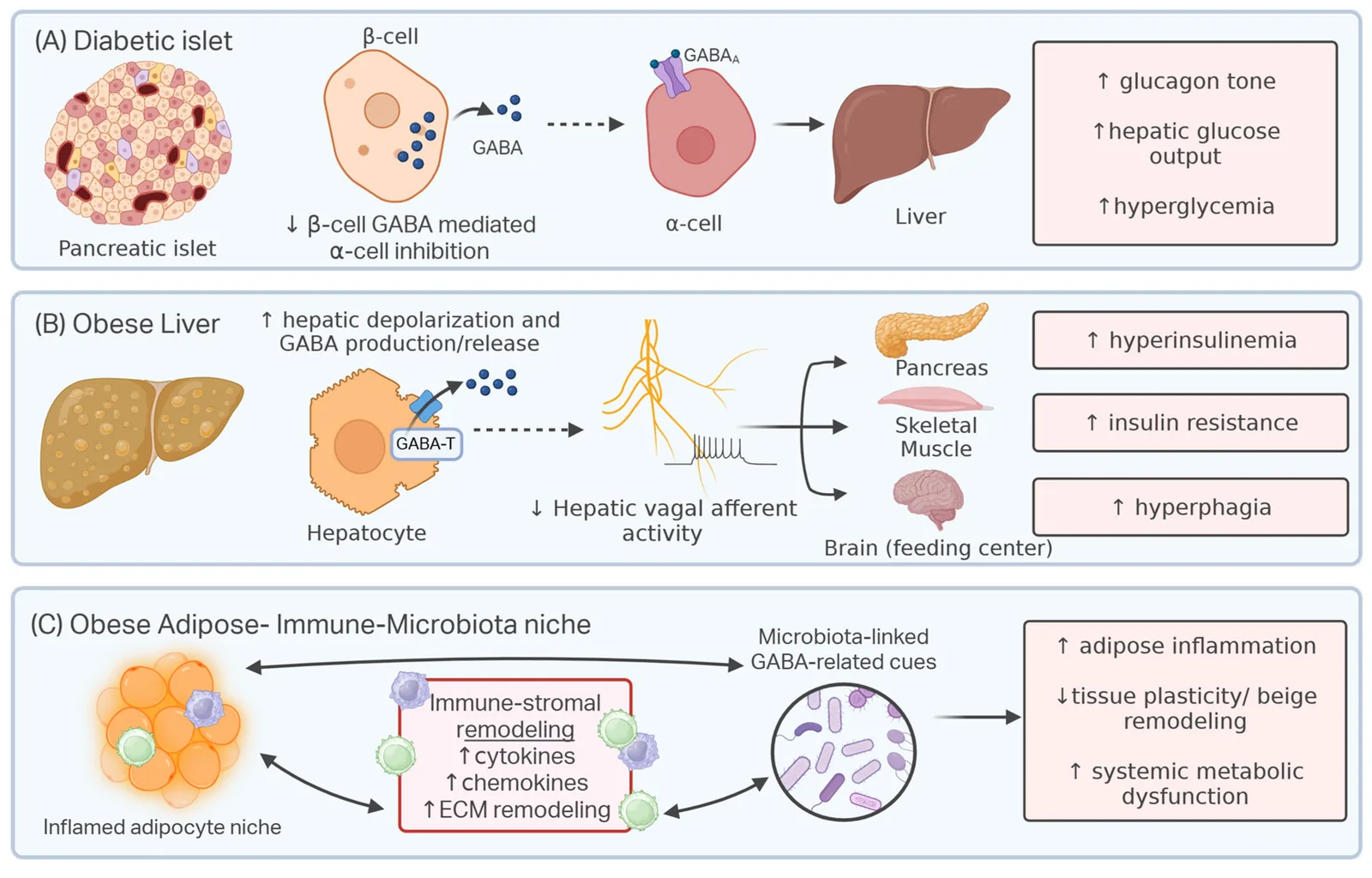
**Disease-state remodeling of GABA-sensitive metabolic circuits.** Obesity and type 2 diabetes mellitus (T2DM) remodel the tissue circuits through which peripheral GABA is produced, sensed and coupled to metabolic output. (**A**) Diabetic islet: impairment of β-cell GABA-mediated α-cell restraint is associated with α-cell dysregulation, increased glucagon tone, increased hepatic glucose output and hyperglycemia. (**B**) Obese liver: hepatocyte depolarization and GABA transaminase (GABA-T)-dependent hepatic GABA output is linked to reduced hepatic vagal afferent activity and systemic outcomes including hyperinsulinemia, insulin resistance and hyperphagia. (**C**) Obese adipose–immune–microbiota niche: inflamed adipocyte signaling, immune–stromal remodeling and microbiota-linked GABA-related signals form a reciprocal niche associated with adipose inflammation, reduced tissue plasticity, impaired beige remodeling and systemic metabolic dysfunction. Solid arrows indicate downstream physiological outputs; dashed arrows indicate proposed or indirect GABA-related signaling; double-headed arrows indicate reciprocal communication. ECM, extracellular matrix; GABA, γ-aminobutyric acid; GABA-T, GABA transaminase; T2DM, type 2 diabetes mellitus.

Against this physiological background, peripheral GABA signaling in obesity and type 2 diabetes mellitus (T2DM) operates within tissues whose metabolic and inflammatory states are remodeled [64,65,66,67]. Altered nutrient flux, impaired insulin action, and chronic inflammatory tone reprogram how local signals are produced, sensed, and coupled to cellular responses across metabolic organs [64,65,66,67]. Consequently, the biological significance of GABA cannot be inferred from changes in pathway activity alone [30,42,43,63]. Rather, its physiological consequence depends on the tissue circuit engaged and on whether that circuit preserves normal metabolic function, transiently offsets tissue stress, or reinforces disease-associated physiology. This context dependence is evident in pancreatic islets, hepatic–neural communication, adipose tissue remodeling, and immune regulation, where GABA-sensitive pathways can assume different functional roles as metabolic disease progresses [30,42,43,63]. Obesity and T2DM therefore reshape peripheral GABA biology not simply by altering GABA abundance, but by changing how tissue-specific pathways are coupled to metabolic function, allowing their effects to remain adaptive, become compensatory, or shift toward maladaptive output (Figure 3).

### 4.1. Diabetic Islets and Impaired GABA-Sensitive Endocrine Restraint

The diabetic islet is not simply a site of β-cell insufficiency, but a remodeled endocrine microenvironment in which local control of hormone output is disrupted [63,68]. In type 2 diabetes mellitus (T2DM), inadequate insulin secretion and impaired insulin action are accompanied by insufficient restraint of glucagon activity, allowing α-cell output to contribute to inappropriate hepatic glucose production and persistent hyperglycemia [69,70]. This failure of glucagon restraint is therefore relevant to the β-cell-to-α-cell GABA pathway that contributes to endocrine coordination under physiological conditions (see Section 3.1) [26,27]. Weakening this pathway would remove one component of local insulin–glucagon coordination and thereby favor inappropriate hepatic glucose production [26,27,63,68].

Human islet studies place this mechanism directly within the context of diabetic endocrine dysfunction. In islets from donors with T2DM, expression of several GABAA receptor subunit genes, including α1, α2, β2, and β3, is reduced, and hormone secretion after GABA receptor modulation is altered [63]. These findings do not identify GABA dysfunction as the sole cause of α-cell dysregulation. Instead, they position GABA as part of a broader failure of intra-islet paracrine control [63,68]. Consistent with this interpretation, paracrine inhibition of α-cell glucagon exocytosis is compromised in human T2DM islets, indicating that abnormal glucagon output reflects impaired local regulation and altered α-cell glucose responsiveness [68]. GABA dysfunction is therefore best understood as a disease-associated defect in endocrine restraint that may contribute to the hormonal imbalance that sustains diabetic hyperglycemia [26,27,63,68].

### 4.2. Obese Liver and Disease-Amplified Hepatic GABA Output

The obese liver provides a clear example of how a physiological GABA-sensitive circuit can be remodeled into a disease-amplified metabolic signal. Obesity-induced hepatocyte depolarization increases GABA release from liver slices, reduces hepatic vagal afferent firing and promotes hyperinsulinemia [42] (see Section 3.2). In obesity, hepatic insulin resistance disrupts the regulation of glucose production and lipid metabolism, while liver-derived signals can transmit hepatocyte stress to extrahepatic tissues [71,72,73]. Within this altered hepatic environment, GABA has emerged as a liver-derived output linked to systemic insulin demand [4,42]. In obese mice, hepatic GABA production increases through a GABA transaminase (GABA-T)-dependent route [4]. Suppressing this pathway, either pharmacologically or through liver-directed GABA-T knockdown, improves multiple features of obesity-associated metabolic dysfunction, including hyperinsulinemia, glucose intolerance, insulin resistance, hyperphagia, weight gain, and impaired peripheral glucose disposal [4]. Human liver data support the relevance of this mechanism, as markers of hepatic GABA production and transport are associated with serum insulin, HOMA-IR, T2DM status, and body mass index in individuals with obesity [4]. These findings position hepatic GABA as a disease-amplified output of the obese liver, rather than as a passive metabolite or a uniformly protective peripheral signal [4,42].

The systemic reach of this hepatic GABA pathway appears to involve both neural and peripheral metabolic routes [4,42]. Obesity-induced hepatocyte depolarization increases GABA release from liver slices, reduces hepatic afferent vagal nerve firing, and promotes hyperinsulinemia [42]. This links the electrical state of hepatocytes to hepatic GABA release and to vagal control of insulin-related physiology [42]. At the same time, liver-directed GABA-T knockdown increases skeletal muscle glucose uptake in obese mice, indicating that altered hepatic GABA metabolism can influence peripheral glucose disposal [4]. This muscle response should not be interpreted as evidence that skeletal muscle is the primary site of GABA dysfunction in obesity. Rather, it shows that a disease-remodeled hepatic GABA pathway can affect an insulin-sensitive tissue that contributes substantially to whole-body glucose clearance [4,74] (see Section 3.5). Together, these studies define hepatic GABA as a liver-derived signal whose metabolic consequence is amplified in obesity [4,42]. The obesity-associated hepatic GABA-output pathway should be distinguished from reports describing metabolic benefits following oral GABA administration. These observations concern different GABA pools and are unlikely to reflect the same anatomical or physiological pathways. The hepatic model concerns increased GABA production and release from depolarized hepatocytes, with downstream effects on hepatic vagal signaling and systemic insulin physiology [4,42]. Oral GABA, by contrast, increases gastrointestinal and circulating exposure and may act through distinct peripheral tissues or signaling routes [6,12,61,75,76]. Beneficial responses to oral GABA do not invalidate the maladaptive hepatic GABA-output model. Rather, the contrasting findings reinforce that the metabolic consequences of peripheral GABA depend on its source, route of availability, and site of action. However, several questions remain unresolved. It remains unclear whether liver-derived GABA acts primarily through hepatic–vagal communication, indirect changes in insulin demand, extrahepatic glucose-disposal pathways, or a combination of these routes [4,42]. Addressing these uncertainties will require studies that pair hepatic GABA production and release with vagal signaling, insulin secretion, feeding behavior, and tissue-specific glucose uptake within the same obesity model.

### 4.3. Obese Adipose Tissue and GABA-Responsive Immune–Metabolic Remodeling

Obesity progressively remodels the cellular architecture and metabolic function of adipose tissue. Adipocyte hypertrophy is accompanied by immune-cell recruitment, stromal reorganization and persistent low-grade inflammation, together compromising the capacity of the tissue to accommodate nutrient excess and maintain metabolic homeostasis [77,78,79,80]. GABA-associated responses in obesity therefore arise within a multicellular niche in which adipocyte, immune and stromal functions are already profoundly altered.

Evidence from experimental models indicates that GABA exposure is associated with changes across several components of this remodeled niche. In high-fat-diet-fed mice, oral GABA improves glucose tolerance and insulin sensitivity while reducing adipocyte hypertrophy, epididymal fat mass and macrophage accumulation [61]. Complementary studies link GABA exposure to reduced stromal-cell-associated monocyte migration and to changes in adipocyte programs governing lipid handling and beige remodeling [55,62]. The convergence of these responses across adipocyte, immune and stromal compartments suggests that GABA-associated remodeling is distributed across the tissue rather than confined to a single cellular target. Whether these effects originate from direct GABA sensing in one or more cell populations or arise secondarily through changes in adiposity, inflammation and intercellular communication remains unclear.

The gut microbiota extends this response beyond the adipose niche by providing a transferable link between oral GABA exposure and beige-associated remodeling. In obese mice, GABA administration alters microbial community composition, whereas transfer of fecal microbiota from treated donors increases thermogenic gene expression in the inguinal white adipose tissue of untreated recipients [43]. The transfer of this feature indicates that part of the adipose response can be conveyed with the altered intestinal microbial community and moves the evidence beyond a simple association between microbiota composition and tissue phenotype. It does not, however, establish that the individual taxa altered by GABA treatment directly drive adipose remodeling or that GABA itself is the transferred mediator. The relevant microbial functions or metabolites, their route of communication with adipose tissue and the host–cell populations through which they act remain unresolved. The emerging model is therefore one in which GABA-associated changes in the intestinal ecosystem may relay signals to adipose tissue, rather than one in which defined bacterial taxa have been shown to cause beige remodeling.

GABA-responsive effects also diverge across adipose depots. In white adipose tissue, GABA exposure is associated with reduced inflammatory remodeling and enhanced beige-associated features [43,55,61,62]. In brown adipose tissue, by contrast, increased GABA accumulation and GABAB-receptor signaling impair thermogenic function during dietary obesity [30]. This depot-specific divergence argues against a uniform adipose response and instead indicates that the metabolic consequences of GABA signaling are shaped by local cellular composition, receptor context and disease state.

Together, these findings position GABA within an adipose–immune–microbiota network in which white adipose remodeling can be influenced by a transferable microbial component, while brown adipose responses are linked more directly to GABA_B_-receptor signaling. The molecular sequence connecting specific microbial functions to defined metabolites, host target cells and whole-tissue remodeling remains unresolved.

## 5. Translational Potential and Therapeutic Strategies

Therapeutic translation of peripheral GABA biology depends on identifying the tissue-specific pathway responsible for a metabolic phenotype rather than indiscriminately increasing or suppressing GABA. Changes in GABA exposure or degradation can produce measurable metabolic effects, but they do not establish the site of action or the physiological pathway engaged [6,75,81]. The relevance of a given intervention is determined by the GABA source, the responding cell population, the disease context, and the intended therapeutic endpoint.

### 5.1. Islet-Directed GABA Strategies

The therapeutic rationale for islet-directed GABA modulation arises from its reported effects on β-cell survival, proliferation, and hormone secretion. These outcomes are not equivalent, and their relevance depends on the underlying islet pathology and the therapeutic endpoint being considered. Experimental models of diabetes or islet injury indicate that GABA administration and related GABAergic interventions can support β-cell survival, proliferation, and secretory function [49,82]. These findings identify a potential role for GABA-sensitive pathways in β-cell maintenance under defined experimental conditions. Proliferation alone, however, is not equivalent to endocrine recovery. Evidence from diet-induced obesity indicates that expansion of β-cell mass does not necessarily correct impaired glucose homeostasis [83]. The relevant endpoint is therefore restoration of glucose-responsive insulin secretion and coordinated glucagon restraint rather than an increase in β-cell number in isolation.

The translational problem differs in T2DM, where insulin resistance and progressive disruption of islet function shape the response to GABA modulation. Islets from donors with T2DM show altered GABAA-receptor subunit expression and abnormal hormone responses following receptor modulation [63]. Receptor composition, residual β-cell function, and α-cell responsiveness may therefore influence whether GABA modulation improves or further perturbs endocrine output. Islet-directed strategies in T2DM should consequently be evaluated against glucose-responsive insulin secretion, glucagon restraint, and glycemic control within the context of established insulin resistance.

A distinct therapeutic problem arises in autoimmune diabetes, where progressive immune-mediated β-cell loss changes both the biological target and the expected endpoint. GAD65 autoantibodies are clinically important markers of type 1 diabetes and latent autoimmune diabetes in adults. Still, this diagnostic association does not establish that increasing GABA exposure can restore endocrine function after immune-mediated β-cell injury [84]. Clinical studies reinforce this distinction. In children with recent-onset type 1 diabetes, oral GABA alone or combined with glutamic acid decarboxylase did not preserve residual insulin secretion as the primary outcome [85]. Long-term GABA treatment likewise did not restore β-cell function in adults with established type 1 diabetes [86]. These studies have therefore not demonstrated preservation or recovery of endocrine function through GABA modulation alone. In this setting, pathway engagement must be linked to the preservation of endogenous insulin secretion and to the control of the underlying immune process.

The therapeutic significance of islet-directed GABA modulation depends on the pathology being addressed. Experimental injury studies emphasize β-cell maintenance, T2DM studies require restoration of coordinated hormone secretion in the setting of insulin resistance, and type 1 diabetes requires preservation of residual endocrine function alongside control of autoimmunity. Disease-specific endpoints are therefore essential for interpreting islet-directed efficacy.

### 5.2. Suppressing Hepatic GABA Output in Obesity

In obesity, the therapeutic target is not peripheral GABA activity, but the obesity-associated increase in hepatic GABA output [4,42]. Liver-directed studies support this direction, showing that suppression of GABA-T-dependent hepatic GABA production improves obesity-associated metabolic dysfunction in mice [4]. This finding establishes hepatic GABA-T as a candidate target for intervention and distinguishes hepatic pathway restraint from strategies that increase systemic GABA exposure [4,42].

Systemic GABA-based interventions can also modify metabolic physiology, but they may act through distinct anatomical pathways [75,76]. Dietary GABA combined with inhibitors of GABA degradation suppresses food intake and promotes weight loss in high-fat-diet-fed mice, primarily through increased plasma GABA availability [75]. In lean mice, high-dose dietary GABA reduces food intake, body-weight gain, and fat accumulation. In contrast, low-dose dietary GABA combined with vigabatrin increases circulating GABA and produces similar anti-obesity effects [76]. These observations show that systemic GABA availability can influence feeding-related physiology, but they do not establish equivalence between circulating GABA elevation and selective suppression of hepatic GABA output [4,75,76].

Evaluation of liver-directed strategies requires a clear distinction between hepatic pathway engagement and downstream metabolic response. Changes in hepatic GABA-T activity and GABA abundance serve as indicators of target engagement. In contrast, hepatic GABA release, hepatocyte membrane potential, and afferent vagal activity define the liver–neural component of the pathway [4,42]. Fasting insulin, HOMA-IR, clamp-derived insulin sensitivity, feeding behavior, and peripheral glucose disposal characterize the resulting systemic response [4,42,75]. Integrating these readouts will be necessary to determine whether an intervention selectively restrains the obesity-associated hepatic GABA pathway rather than merely altering circulating GABA or whole-body metabolism. This level of resolution will determine whether hepatic GABA-T can be advanced as a mechanism-based target for obesity-associated insulin resistance [4,42,87].

### 5.3. Therapeutic Opportunities in Adipose GABA Signaling

The therapeutic opportunity in adipose GABA signaling lies in separating adaptive from maladaptive tissue responses. In white adipose tissue, GABA-associated effects converge on the preservation of tissue plasticity and attenuation of inflammatory remodeling [43,55,61,62]. In brown adipose tissue, by contrast, GABA_B_-receptor signaling suppresses thermogenic function during obesity [30]. This functional divergence argues against generalized elevation of peripheral GABA and instead supports a depot-directed therapeutic strategy.

In white adipose tissue, the principal objective would be to preserve tissue competence during nutrient excess. GABA-responsive processes associated with beige potential, lipid handling and immune–stromal balance offer a means of sustaining metabolic flexibility and limiting the transition towards hypertrophic and inflammatory dysfunction [43,55,61,62]. The therapeutic value of this approach would derive less from reducing adipose mass in isolation than from maintaining the capacity of white adipose tissue to store and dissipate energy without amplifying systemic metabolic stress. The microbiota-associated component of this response broadens the therapeutic landscape beyond the adipose depot itself. The transfer of selected thermogenic features with fecal microbiota from GABA-treated donors indicates that bioactive functions generated within the intestinal ecosystem can influence white-adipose plasticity [43]. Modulation of these microbial functions could therefore complement adipose-directed approaches by engaging adaptive tissue remodeling indirectly.

In brown adipose tissue, the therapeutic direction is reversed. Restraint of GABA_B_-receptor signaling could preserve thermogenic output and energy expenditure during obesity [30]. Adipose GABA biology therefore supports a dual therapeutic logic: reinforce adaptive function in white adipose tissue while relieving inhibitory GABA signaling in brown adipose depots.

### 5.4. Barriers to Translation

Current evidence supports the role of peripheral GABA signaling in metabolic regulation; however, important limitations remain. Most mechanistic evidence derived from animal models and cell-based systems, whereas human studies remain comparatively limited. Consequently, the principal barrier to translation is not the absence of metabolic phenotypes but the difficulty of linking those phenotypes to a defined GABA source, accessible tissue, responding cell population, and engaged pathway. Peripheral GABA exposure may arise from endogenous synthesis, dietary intake, or microbial metabolism, but these routes do not necessarily yield the same biologically relevant signal. Rather than indicating where GABA acts, measures of circulating or tissue GABA primarily reflect exposure or abundance. Their translational value depends on establishing that a specific GABA pool reaches an accessible tissue compartment, engages an identifiable cellular pathway, and produces a measurable physiological response. Without this level of resolution, changes in peripheral GABA cannot be interpreted as evidence of therapeutic target engagement. Plasma GABA concentrations and total tissue GABA content should be interpreted as indicators of exposure or overall abundance rather than as evidence that a specific GABA signaling pathway has been activated within a particular cell population.

This distinction is particularly important because interventions that modify GABA exposure can simultaneously alter food intake, body weight, insulin demand, microbial composition, and inflammatory tone [5,43,55,61,62]. Each of these variables can influence glucose regulation and adipose metabolism independently of direct GABA signaling [77,78,79,80,88,89,90]. For example, a feeding schedule can modify weight gain and glucose control even when caloric intake is comparable [88]. In contrast, differences in microbial-community function can alter dietary energy harvest and adipose accumulation [89]. Intestinal permeability and exposure to microbial inflammatory products provide an additional route through which microbial changes can affect insulin sensitivity and body-weight regulation [90]. Metabolic improvement following a GABA-associated intervention cannot be assigned to GABA solely based on concurrent changes in circulating GABA or tissue abundance. Studies must distinguish direct pathway engagement from secondary effects mediated by energy balance, adiposity, microbial ecology, or inflammation.

Human supplementation studies further illustrate why systemic exposure cannot be equated with disease-specific efficacy. Prediabetes represents an early dysglycemic state in which insulin resistance and compensatory β-cell responses precede established type 2 diabetes mellitus. In adults with prediabetes, however, GABA supplementation did not improve the primary postprandial glucose endpoint compared with placebo [81]. This finding indicates that increased peripheral availability alone is insufficient to produce metabolic benefit, even during an early stage of dysglycemia. A similar lack of disease-modifying efficacy has been observed in type 1 diabetes, a setting characterized by substantial β-cell loss. In this context, GABA-based interventions have not preserved or restored β-cell function as a primary outcome [85,86]. Taken together, these studies do not negate the biological activity of GABA but indicate that increased exposure alone does not necessarily translate into clinically meaningful benefits. Therapeutic relevance depends on whether GABA engages the appropriate target, within the appropriate tissue and disease context, and produces a physiological response matched to the underlying mechanism.

Disease stage further determines whether a GABA-responsive pathway remains therapeutically accessible. Progressive metabolic remodeling alters receptor availability, cellular responsiveness, and tissue architecture, such that pathways supporting adaptive regulation in early disease may become dysregulated or functionally inaccessible as pathology progresses. Therapeutic interpretation requires biomarkers that distinguish preserved pathways from those that have become maladaptive or are no longer responsive to intervention. Such biomarkers will be essential for selecting patients in whom modulation of a specific GABA-sensitive pathway remains biologically meaningful.

A related obstacle is the absence of validated tissue-specific markers of pathway engagement. Changes in circulating GABA, glucose tolerance, or inflammatory mediators cannot, by themselves, establish that the intended receptor, transporter, or metabolic pathway has been modulated in the relevant organ. Mechanism-matched biomarkers will be required for each therapeutic context. In pancreatic islets, these might include coordinated changes in insulin and glucagon secretion together with measures of residual β-cell function and α-cell receptor status [26,27,63]. In the liver, informative measures may include hepatic GABA abundance, GABA transaminase activity, liver–neural output, and changes in fasting insulin demand [4,42]. In adipose tissue, relevant endpoints may include immune-cell composition, stromal-cell behavior, thermogenic or beige-remodeling programs, and depot-specific tissue responses [30,43,61,62]. Such biomarkers would provide the necessary link between intervention, tissue engagement, and systemic metabolic outcome.

Dose, timing, and route of administration also require greater resolution. Oral GABA, GABA-producing microorganisms, receptor-directed compounds, and GABA metabolism inhibitors are mechanistically distinct interventions. They differ in pharmacokinetics, duration of exposure, tissue distribution, and the probability of engaging both central and peripheral pathways. Their effects may also depend on feeding state, circadian timing, and disease stage. Dose escalation based only on circulating GABA may therefore fail to identify the exposure needed within the target tissue and could increase off-target effects without improving efficacy. Translational studies should determine the minimum exposure required for pathway engagement and establish whether sustained, intermittent, or meal-related administration best matches the underlying mechanism.

Safety will likewise depend on the pathway selected. Broad enhancement of GABA signaling is unlikely to reproduce the consequences of selectively inhibiting hepatic GABA production, modulating a defined receptor population, or increasing microbial GABA output. The contrasting effects observed across pancreatic, hepatic, and adipose compartments indicate that systemic activation or inhibition could improve one pathway while impairing another [30,43,63]. Therapeutic development should therefore prioritize tissue-restricted delivery, receptor or pathway selectivity, or interventions that modify local GABA metabolism without broadly altering signaling elsewhere. Longer-term studies will also be needed to determine whether metabolic improvement can be maintained without adverse effects on endocrine regulation, thermogenesis, immune function, or neural communication.

Collectively, these challenges indicate that successful translation will depend less on manipulating peripheral GABA abundance than on defining the precise pathway through which GABA acts in a given disease context. Future studies should therefore prioritize the resolution of GABA source, tissue access, the responding cell population, and downstream physiological consequences within the same experimental framework. Such pathway-level resolution will be essential for distinguishing causal therapeutic mechanisms from associated metabolic phenotypes. It will also guide the investigation of unresolved questions summarized in Box 1.

Box 1 **Outstanding questions in peripheral GABA biology and metabolic disease.**

- What is the biologically active source of peripheral GABA within pancreatic, hepatic and adipose tissues, and does this source change during metabolic disease?
- How does circulating or intracellular GABA abundance relate to local extracellular availability and pathway engagement within individual tissues?
- Which cell populations and molecular pathways determine whether peripheral GABA responses are adaptive or maladaptive?
- To what extent are the mechanisms identified in animal models and cell-based systems conserved in human metabolic disease?
- Can tissue-specific biomarkers distinguish direct GABA pathway engagement from secondary changes in food intake, body weight, microbiota composition or inflammatory tone?
- At what disease stage, and in which patient populations, could selective modulation of peripheral GABA signaling provide metabolic benefit without producing opposing effects in other peripheral tissues?

## 6. Conclusions and Outlook

Peripheral GABA biology has moved beyond the question of whether GABA acts outside the nervous system. The central challenge now is to determine when peripheral GABA signaling supports adaptive metabolic regulation, reflects compensatory remodeling, or contributes to disease-amplified dysfunction. This distinction is complicated by the multiple sources of peripheral GABA and by disease-related changes in the tissues and cell populations that receive and interpret these signals. Host metabolism, dietary exposure, and microbial production can each contribute to peripheral GABA availability [6,14,18,34]. However, these sources do not necessarily generate equivalent receptor-accessible signals or physiological consequences. In obesity and type 2 diabetes mellitus (T2DM), impaired insulin action and chronic inflammatory remodeling reshape the cellular environments in which GABA-sensitive pathways operate [65,67].

Bulk GABA abundance should therefore be regarded as a starting point rather than a translational endpoint. Changes in circulating or tissue GABA may reflect altered exposure, metabolism, compartmentalization, or pathway engagement, and concentration measurements alone cannot distinguish among these possibilities. We envision a shift from abundance-based measurements towards defining the biological pathways through which GABA acts. Achieving this resolution will require approaches that integrate GABA source, tissue access, responsive cell populations, and downstream physiological consequences within a single experimental framework. Isotope tracing, spatial metabolomics, single-cell and spatial profiling, microbiome functional analysis, and tissue-selective perturbation will be particularly important for resolving these relationships [91,92,93,94]. We also anticipate that human-relevant models will become increasingly important for determining whether specific GABA-sensitive pathways should be preserved, restrained, or redirected. A pathway that supports adaptation in one tissue or stage of disease may become ineffective or maladaptive in another, and systemic GABA exposure alone does not reliably predict metabolic or endocrine benefit [81,85,86]. Translation will therefore depend on pathway-specific biomarkers, evidence of target engagement, and physiological endpoints matched to the proposed mechanism.

Such mechanistic resolution will move peripheral GABA research beyond documenting the presence of a familiar molecule towards defining how GABA-sensitive communication contributes to metabolic adaptation, maladaptation and therapeutic opportunity.

## Figures and Tables

**Table 1:** **Representative evidence for organ-specific actions of peripheral GABA.**

Tissue or Compartment	Experimental Model	GABA Source or Intervention	Principal Tissue-Linked Outcome	Key Limitation	Refs.
**Pancreatic** **islet: α-cell regulation**	Rat islets and isolated α-cells	Endogenous β-cell-derived GABA; intra-islet insulin modulation of α-cell GABA_A_ receptors	GABA contributes to glucose-dependent restraint of glucagon secretion; insulin enhances α-cell GABA_A_-receptor trafficking and signaling	Mechanistic evidence is derived mainly from rodent islet systems; the magnitude of this pathway in human islets remains less clearly defined	[26,27]
**Pancreatic islet: human β-cells**	Isolated human β-cells and human islet preparations	GABA_A_-receptor activation	Human β-cells express functional high-affinity GABA_A_ receptors; receptor activation can be depolarizing and can influence insulin secretion	The response depends on cellular chloride gradients, receptor composition, and experimental conditions	[28,29]
**Liver**	Liver slices and in vivo studies involving manipulation of hepatocyte membrane potential and hepatic vagal signaling	Hepatocyte-derived GABA release	Hepatic GABA release is linked to reduced hepatic vagal afferent activity and changes in circulating insulin and insulin sensitivity	The relative contributions of direct hepatic–vagal signaling and secondary endocrine effects remain incompletely separated	[42]
**White adipose tissue–microbiota axis**	High-fat-diet-fed mice and fecal microbiota-transfer experiments	Oral GABA and transfer of microbiota from GABA-treated donors	GABA treatment promoted inguinal white-to-beige adipose remodeling; microbiota transfer reproduced features of the adipose phenotype	The study supports microbiota involvement but does not establish whether GABA itself or another microbiota-dependent signal mediates the adipose response	[43]
**Peripheral blood immune cells**	Human peripheral blood mononuclear cells and purified CD4^+^ T cells	Exogenous GABA and GABA-receptor modulation	GABA reduced the release of several inflammatory cytokines	Direct immune-cell responsiveness is demonstrated, but a causal contribution to whole-body metabolic regulation has not been established	[3]
**Macrophages**	Cellular and mouse macrophage models	GABA transport and GABA-associated metabolic signaling	GABA-sensitive pathways regulated IL-1β production and inflammatory output	The direction and magnitude of the response depend on transporter activity, cellular metabolism, and macrophage activation state	[44,45]
**Skeletal muscle: metabolic incorporation**	Mice receiving dietary GABA	Oral GABA	Dietary GABA increased skeletal-muscle homocarnosine, demonstrating incorporation into a GABA-containing imidazole dipeptide	The finding establishes metabolic incorporation but not direct regulation of muscle insulin signaling, glucose uptake, or mitochondrial function	[35]
**Skeletal muscle: regeneration**	Diabetic mice with impaired muscle repair	Oral GABA	GABA administration improved muscle-regeneration outcomes	The directly responding cell population and the receptor-dependent or receptor-independent mechanism remain unresolved	[46]
**Gut microbiota**	Mice treated with GABA-producing lactobacilli or microbiota-directed interventions	GABA-producing bacterial strains; fructooligosaccharide and enzyme-based microbiota manipulation	Interventions altered intestinal or tissue GABA-related pools and were accompanied by changes in selected host phenotypes	The precise microbial source, route of host access, and contribution of GABA relative to other microbiota-derived signals remain incompletely resolved	[5,13]

## Data Availability

No new data were created or analyzed in this study. Data sharing is not applicable to this article.
